# Different ways to forget: Electrophysiological mechanisms underlying item-method directed forgetting of angry and neutral faces

**DOI:** 10.3389/fnbeh.2022.957227

**Published:** 2022-09-15

**Authors:** Johanna Kissler, Anne Hauswald

**Affiliations:** ^1^Department of Psychology, Bielefeld University, Bielefeld, Germany; ^2^Center of Cognitive Interaction Technology, Bielefeld University, Bielefeld, Germany; ^3^Department of Psychology, University of Salzburg, Salzburg, Austria; ^4^Centre for Cognitive Neuroscience, University of Salzburg, Salzburg, Austria

**Keywords:** emotion, memory, facial expression, directed forgetting, event-related potential, electroencephalography

## Abstract

Emotional stimuli, including faces, receive preferential processing and are consequently better remembered than neutral stimuli. Therefore, they may also be more resistant to intentional forgetting. The present study investigates the behavioral and electrophysiological consequences of instructions to selectively remember or forget angry and neutral faces. In an item-method directed forgetting experiment, angry and neutral faces were randomly presented to 25 student participants (4 males). Each face was followed by an instruction to either forget or remember it and the participants’ EEG was recorded. Later, recognition memory was unexpectedly tested for all items. Behaviorally, both hit and false alarm rates were higher for angry alike than for neutral faces. Directed forgetting occurred for neutral and angry faces as reflected in a reduction of both recognition accuracy and response bias. Event-related potentials revealed a larger late positive potential (LPP, 450 – 700 ms) for angry than for neutral faces during face presentation and, in line with selective rehearsal of remember items, a larger LPP following remember than forget cues. Forget cues generally elicited a larger frontal N2 (280 – 400 ms) than remember cues, in line with the forget instruction eliciting conflict monitoring and inhibition. Selectively following angry faces, a larger cue-evoked P2 (180 – 280 ms) was observed. Notably, forget cues following angry faces elicited a larger late frontal positivity (450 - 700 ms) potentially signaling conflict resolution. Thus, whereas both angry and neutral faces are subject to directed forgetting, on a neural level, different mechanisms underlie the effect. While directed forgetting for neutral faces may be achieved primarily by selective rehearsal, directed forgetting of angry faces involves an additional late frontal positivity, likely reflecting higher cognitive demands imposed by forgetting angry faces.

## Introduction

Emotional facial expressions are highly relevant social signals. Angry expressions, in particular, convey a potential threat to the individual and rapidly mobilize defensive reactions (Dimberg and Öhman, 1996). Angry faces are also detected faster in multi-stimulus visual displays (Hansen and Hansen, 1988; Ohman et al., 2001), are less susceptible to the attentional blink (Maratos et al., 2008), affect spatial orientating already pre-attentively (Mogg and Bradley, 1999), and capture the brain’s attentional resources at multiple processing stages (Schupp et al., 2004; Holmes et al., 2009). Electrophysiologically, both the occipito-temporal early posterior negativity (EPN) and the parietal late positive potential (LPP) are enhanced during viewing of angry faces compared with happy or neutral ones (Schupp et al., 2004). Larger late parietal positivities often predict better subsequent memory for emotional stimuli (Dolcos and Cabeza, 2002), in line with findings of generally better memory for emotional events and stimuli (for review see Kensinger and Schacter, 2008), including faces with emotional expressions (Johansson et al., 2004).

While it is often useful to remember an emotional event, this may not hold for every emotional encounter. For instance, remembering the new neighbor’s angry face may be important, because it may be predictive of this neighbor’s character in general and future encounters with this person. On the other hand, the anger on the neighbor’s face might have been a mere coincidence, not worth remembering, because this neighbor might in general be a very charming person. This example illustrates the need to explicitly control memory encoding and to decide whether or not a memory should be formed and retained, although this may be sometimes difficult.

One experimental paradigm that tests for explicit control of memory is the directed forgetting paradigm (DF). In its item-method, stimuli are presented individually and each is followed by an instruction to either remember or forget it. When later memory for all stimuli is unexpectedly tested, regardless of their previous instruction, not surprisingly, the previously to-be-remembered items are recalled or recognized better than the to-be-forgotten items (Weiner, 1968; Weiner and Reed, 1969; Bjork, 1970). This effect is thought to arise either because after the cue is presented only remember items are rehearsed further, which is referred to as the selective rehearsal account of directed forgetting (Basden et al., 1993; Basden and Basden, 1996; MacLeod, 1999), or because forget items are somehow inhibited (Hourihan and Taylor, 2006; Fawcett and Taylor, 2008a,b). These two mechanisms are not mutually exclusive, and evidence for both has been put forward. Although it is not entirely clear whether an explicit instruction to forget really represents the most effective way to make people forget previously encountered information (Zwissler et al., 2015; Schindler and Kissler, 2018; Gao et al., 2019; see also Wegner, 1994), clearly, in everyday life markers of mnemonic relevance can arise after information has been encountered. Likewise, item-method DF is a much-used experimental paradigm for which at least subsequent attentional inhibition is well-established (Fawcett and Taylor, 2008a,b) and whose neurocognitive mechanisms need to be specified further.

Previous research has repeatedly shown that item-method DF can be modulated by stimulus emotion. For instance, it has been reported to be smaller for short phrases describing negative rather than neutral events (Lee and Hsu, 2013). Likewise, for highly emotionally arousing pictures, reduced (Nowicka et al., 2011; Zwissler et al., 2012), or even absent (Hauswald et al., 2011; Zwissler et al., 2011) item-method DF has been reported. On the other hand, Yang et al. (2012) reported item-method DF effects of similar magnitude for negative and neutral pictures, when negative and neutral pictures were matched for arousal which was not the case in the studies by Hauswald et al. (2011). For emotional words, item-method DF effects have been also found to be reduced (Bailey and Chapman, 2012; Gallant and Dyson, 2016; Alfonso and Menor, 2021), although some evidence indicates that they can even be larger than for neutral ones (Brandt et al., 2013). A recent meta-analysis on emotion modulation of item-method DF suggests that across studies, emotional stimuli diminish the effect by about 4%, although there is considerable variability across studies (Hall et al., 2021).

Regarding item-method DF of faces with emotional expressions, Quinlan and Taylor (2014) found equivalent DF effects for happy, angry and neutral faces across two experiments that varied stimulus exposure time. Tay and Yang (2017), by contrast, reported angry faces to be more resistant to DF than happy faces. Finally, Corenblum et al. (2020) recently reported DF effects for happy and neutral, but not for sad faces. Thus, current behavioral data on effects of facial expressions on item-method DF suggest no reduction by happy expressions whereas the evidence is inconsistent regarding angry expressions. Together, Tay and Yang’s (2017) and Corenblum et al. (2020) findings indicate that faces with negative expressions may be harder to forget than neutral of positive expressions which would be in line with many other findings on DF of emotionally negative stimuli (see Hall et al., 2021). On the other hand, two experiments by Quinlan and Taylor (2014) challenge this assumption, calling for further research on DF of negative, and particularly angry faces.

Regarding the neural mechanisms of item-method DF, studies have revealed more pronounced parietal activity in response to the remember instruction (Paz-Caballero et al., 2004; Hauswald et al., 2011; van Hooff and Ford, 2011) which seems in line with the selective rehearsal account. By contrast, specific frontal activities elicited by the forget cue (Paz-Caballero et al., 2004; Wylie et al., 2008; Hsieh et al., 2009; Hauswald et al., 2011; van Hooff and Ford, 2011; Brandt et al., 2013; Rizio and Dennis, 2013; Alfonso and Menor, 2021) are often interpreted as reflecting inhibitory mechanisms of item-method directed forgetting. In fact, frontal brain activities (Wylie et al., 2008), in the EEG often late ERP positivities (from around 400 ms), have been reported to correlate with the magnitude of the DF effect (Hauswald et al., 2011; Alfonso and Menor, 2021) and to differentiate intentionally forgotten items from incidentally forgotten ones in subsequent memory analyses (van Hooff and Ford, 2011).

As stated above, the greater difficulty of forgetting emotional material may be due to the fact that emotional stimuli are processed more deeply during initial presentation, before the memory cue appears. In ERP studies of item-method DF, this is reflected in higher item-related late positive potentials (LPP) during presentation of emotional items which for pictures have a centro-parietal distribution (see e.g., Hauswald et al., 2011). Accordingly, in their study on directed forgetting of emotional words, Brandt et al. (2013) observed larger LPPs during presentation of emotional words. Although their word-evoked positive potentials had a predominantly frontal distribution (perhaps due to the verbal material used), the data is conceptually consistent with evidence of better incidental memory encoding of emotional stimuli (Dolcos and Cabeza, 2002). During cue presentation, Brandt et al. (2013) found a frontal positivity to be larger for forget than remember cues, replicating other previous research (Paz-Caballero et al., 2004; Hauswald et al., 2011). The amplitude of this frontal positivity was not modulated by item emotion. Further, remember cues induced larger LPPs than forget cues and this effect was larger for remember cues following emotional words. Thus, in the study by Brandt and colleagues stronger incidental encoding during word presentation and more pronounced post-cue selective rehearsal of emotional “remember” words may have given rise to the larger DF effect for emotional words, effectively amounting to a “directed remembering” effect. Alfonso and Menor (2021) likewise report a larger late frontal positivity during presentation of emotional rather than neutral words. In response to the memory cue, these authors also report a larger posterior positivity for remember than for forget cues which correlated with better word recognition. This parietal positivity was larger for cues following negative rather than neutral words. For forget cues, this study found a frontally dominant, but widely distributed late positivity which was more pronounced for forget cues following neutral than negative words. Assuming that the R-cue related positivities reflect rehearsal and the late F-cue related positivity reflects inhibition, in the Alfonso and Menor (2021) study, both selective rehearsal and active inhibition could have contributed to the behavioral pattern. DF occurred for both negative and neutral words, but was reduced in magnitude for negative words.

In addition to relatively late-occurring frontal and parietal positivities, cue-driven modulations of earlier ERPs such as the frontal P2/N2 component have also been observed in item-method DF paradigms (e.g., Gao et al., 2016; Schindler and Kissler, 2018). Specifically, Gao et al. (2016) suggested a larger frontal P2 to index attention allocation to TBR cues. A subsequent larger N2 for F-cues has been interpreted as reflecting inhibition and information discarding, consistent with this component’s role in the stop signal task of motor inhibition (e.g., Schmajuk et al., 2006; Nigbur et al., 2015) or memory inhibition in the Think-No Think task (Bergström et al., 2009; Mecklinger et al., 2009). Regarding further modulation by preceding emotional content, Yang et al. (2012) in their study on item-method DF of emotional and neutral pictures reported both larger N2 elicited by F-cues than by R-cues and larger N2 for F-cues following negative than F-cues following neutral pictures. Comparing item-method DF in healthy people and schizophrenia patients, Patrick et al. (2015) also found that in healthy adults, F-cues elicited a larger N2 than did R-cues, although they did not observe any effect of the emotional content (negative or neutral) of the words preceding the cues.

Overall, extant data seem compatible with the view that the item-method DF effect can arise both *via* selective rehearsal of remember items and active inhibition of forget items. The magnitude of the net effect and its modulation by emotion might be determined by the relative contribution of either process. Because words may lend themselves more to selective rehearsal in working memory than does pictorial material, capitalizing both on phonological and visuo-spatial rehearsal (Baddeley, 2003; Brandt et al., 2013), larger DF effects for emotional stimuli might by more likely for words than for pictorial stimuli. For pictures, by contrast, more pronounced incidental encoding of negatively arousing pictures already during initial stimulus presentation may counteract directed forgetting (e.g., Hauswald et al., 2011). Accordingly, the higher the parietal positivity elicited by the pictures themselves, the smaller the DF effect and the larger the frontal positive amplitude elicited by F-cues, the bigger the effect of directed forgetting (Hauswald et al., 2011). So far, several studies found a relatively late frontal positivity to be larger in response to forget than remember cues (e.g., Paz-Caballero et al., 2004; Hauswald et al., 2011; Brandt et al., 2013; Alfonso and Menor, 2021), but its functional relevance is not fully clarified. Moreover, some studies suggested the frontal N2 as a correlate of inhibition in item-method DF.

Faces are a very salient, socially relevant and emotionally evocative stimulus class, and humans often need to selectively memorize them to distinguish between individuals and affective states. Still, so far only few studies have examined directed forgetting for emotional faces and to the best of our knowledge none has simultaneously collected neurophysiology data. Therefore, we investigate the behavioral pattern and electrophysiological correlates of directed forgetting of angry and neutral faces. The experimental set-up and analysis closely parallel our previous report using negatively arousing and neutral un-arousing picture stimuli (Hauswald et al., 2011) from the International Affective Picture System (IAPS, Lang et al., 1997). In an old-new recognition memory paradigm, we test whether item-method DF for angry faces will behaviorally differ from item-method DF for neutral faces and examine electrophysiological mechanisms underlying the DF effect (or its possible absence) for neutral and angry faces. We analyze stimulus-evoked and cue-evoked ERPs in the encoding phase of the experiment and aim to relate them correlatively to recognition performance. We focus on the face-evoked LPP as well as the cue-evoked frontal P2, N2 and late frontal and late parietal positivities. Based on previous research, we expect larger face-evoked LPP in response to angry than neutral faces. Regrading cue-evoked early frontal ERPs, we also expect larger P2 elicited by R-cues than by F-cues, but lager N2 in response to F-cues than in response to R-cues. Regarding cue-evoked late positivities, we expect a larger frontal late positivity elicited by the F-cue and a larger parietal late positivity elicited by the R-cue. Given that there is no previous ERP research on item-method DF of emotional faces, we have no clear hypothesis on further modulations of cue-evoked ERPs by angry versus neutral faces but will analyze these *via* statistical tests.

## Materials and methods

### Participants

In total, 25 students (21 female) from the University of Konstanz, Germany (mean age: 24.2) participated in the experiment. The participants provided informed consent and received course credit or a financial compensation of 15 €.

### Stimuli, procedure, design

**FIGURE 1:**
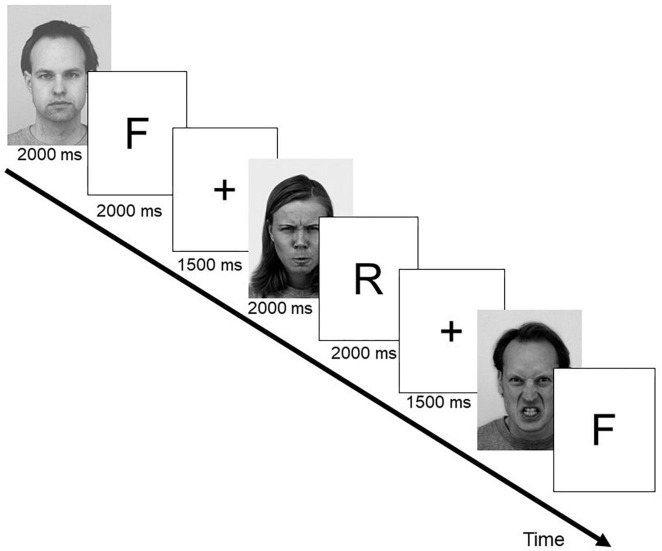
Exemplary trial sequence and timing parameters for the encoding phase of the directed forgetting experiment. F represents a forget cue, denoting the previous face as “to-be-forgotten,” R indicates a remember cue denoting the previous face as to “to-be-remembered.” Example faces shown here are from the KDEF database (Lundqvist et al., 1998). Shown are KDEF #AM10ANS,#AF19ANS, and #AM02NES.

The experimental design mirrored the one used by Hauswald et al. (2011). Stimulation was run under Presentation (Neurobehavioral Systems, Albany, United States). A set of 240 black-and-white pictures of faces (50% male, 50% female) selected from different published affective faces databases was used. It included 130 photographs from the Park Aging Mind Laboratory (Minear and Park, 2004), 46 from the Karolinska Directed Emotional Faces (KDEF, Lundqvist et al., 1998), 36 from the NimStim (MacArthur Foundation Research Network on Early Experience and Brain Development), 23 from a stimulus set developed at the University of Münster, Germany, and five photographs from the AR Face Database (Martinez and Benavente, 1998). 120 photographs showed faces with neutral expressions, 120 had angry expressions. Half of the photographs (60 neutral expressions, 60 angry expressions) were presented in the learning phase and were randomly assigned either a “forget” or a “remember” instruction. The remaining photographs served as distracters in the recognition task. Figure 1 illustrates the encoding phase of the experiment.

During encoding, photographs displaying faces with neutral or angry expressions were presented individually in a pseudo-random consecutive sequence. Each face was shown for 2000 ms each and was directly followed by either a “forget” (F-cue, indicated by VVV for German “vergessen”) or a “remember”-cue (R-cue, indicated by MMM for German “merken”) presented for another 2000 ms. Hereafter, a fixation cross was shown for 1500 ms before the next face was presented. Half of the neutral and half of the angry expressions were followed by an F-cue. Faces from the remaining halves were followed by an R-cue. Participants were instructed to memorize the faces followed by the R-cue and to forget those followed by the F-cue. The faces were shown in three blocks of 40 consecutive face-cue pairs, after each of which a short break (7 s), where participants could blink, was given. After the encoding phase, the participants performed for 5 min a speeded digit cancelation task (d2, Brickenkamp, 1994) as a distracter task. In the subsequent recognition test, all 120 pictures from the encoding phase and the remaining 120 distracter pictures were presented in random order for 300 ms each. Participants had to perform an old-new recognition test, regardless of the initial forget or remember instructions. Participants were instructed to react as quickly and accurately as possible (see also Hauswald and Kissler, 2008). Reaction time data were corrected for outliers (exceeding ± 2 standard deviations).

### EEG recording

The EEG was recorded from 65 Ag/AgCl electrodes using Neuroscan (Scan, SynAmps, Compumedics, El Paso, United States) soft- and hardware. During recording, electrodes were referenced to Cz. Impedances were kept below 5 kΩ. Data were acquired with a sampling rate of 500 Hz and online filters of DC to 100 Hz. Prior to the experiment vertical, horizontal, and blink-related eye movements were recorded from each participant for later eye artifact correction of the experimental EEG data.

### Data analysis

The recorded EEG data was pre-processed using the Brain Electrical Source Analysis package (Besa^®^, MEGIS Software GmbH, Gräfeling, Germany). Offline, data were re-referenced to an average reference. Data were corrected for eye movements, using individual calibrations and a topographic correction algorithm implemented in BESA (Ille et al., 2002) and any remaining large artifacts were rejected (EEG > 220 μV). For statistical analysis of neural activity, the artifact corrected data were band-pass filtered from 0.3 to 30 Hz, split into epochs (−100 ms – 1000 ms), baseline corrected using a 100 ms pre-stimulus epoch, and averaged. ERPs were aligned to face onset and cue onset. EEG data visualization and statistical analysis was performed using ElectroMagnetic EncephaloGraphy Software EMEGs^®^, www.emegs.de (Peyk et al., 2011). Statistical analysis was conducted on average ERP activity within electrode groups of interest defined based on our previous research. Time-windows of interest were defined based on a combination of visual inspection and previous research.

#### Face presentation

A group of 8 electrodes (C1, C2, CPz, CP3, CP4, Pz, P1, P2) was extracted for statistical analysis of late positive potentials elicited by the faces in a time window from 450 to 700 ms after picture onset. This group of electrodes and a similar time-window (450–900 ms) had already been used in Hauswald et al. (2011).

#### Memory cue presentation

##### P2/N2

The frontal P2 and N2 components were identified and analyzed from an averaged group of 9 electrodes (AFz, AF3, AF4, Fz, F1, F2, FCz, FC1, FC2) in a time-window from 180 to 280 ms (P2) and 280 to 400 ms (N2). A similar group of electrodes had also been used in Schindler and Kissler (2018).

##### Late parietal positivity

In line with the literature, an enhanced parietal positivity in response to R-cues between 350 and 550 ms after cue onset was analyzed using the same parietal electrode group as for face presentation. The same approach was used in Hauswald et al. (2011), where the analyzed time-window for the parietal cue-evoked activity extended from 400 to 500 ms.

##### Late frontal positivity

Moreover, an increased positivity in response to F-cues following neutral faces was identified and analyzed at the same group of 9 frontal sensors as the P2/N2 (AFz, AF3, AF4, Fz, F1, F2, FCz, FC1, FC2) from 450 ms after cue onset until 700 ms after cue onset. This group of electrodes had already been used in Hauswald et al. (2011) where the time-window used was 450 ms to 700 ms.

### Statistical analysis

All statistical calculations were done in JASP (Love et al., 2019) and emegs3.1 (Peyk et al., 2011). *Post-hoc*, ANOVAs involving multiple factors were broken down into smaller ANOVAs. Pairwise comparisons were calculated using *t*-tests. An alpha level of 0.05 was used for all calculations. Effect sizes are reported using partial eta squared (η_*p*_^2^). According to Cohen (1992), effects are interpreted as small (η*_*p*_^2^* > 0.02), medium (>0.13), or large (>0.26). *Post-hoc* power analyses were computed using G* Power 3.1.9.7 (Faul et al., 2007) using effect size specification according to Cohen.

#### Behavioral data

Repeated-measures ANOVAs were calculated for recognition rates and reaction times with the within-factors recognition (hit, miss), instruction (F-item ∼ forget items, R-item ∼ remember items) and valence (angry, neutral) for previously presented items and with the factors response (correct rejection, false alarm) and valence (angry, neutral) for distractor items.

Furthermore, discrimination index (Pr = hits - false alarms) and response bias (Br = false alarms/[1 - Pr]) were calculated according to Snodgrass and Corwin’s (1988) two-high-threshold model. Br values of 0.5 indicate no response bias, while higher values indicate a liberal and lower values a conservative response strategy.

#### Electrophysiological data

##### Picture presentation

A repeated-measures ANOVA with the factor valence (angry, neutral) was used to assess parietal brain activity reflecting spontaneous attention capture and automatic encoding between 450 and 700 ms after picture onset comparing across conditions average event-related activity within the parietal electrode group specified above.

##### Cue presentation

To investigate cue-related parietal brain activity, an ANOVA with the within-factors instruction (F-cue, R-cue) and valence (angry, neutral) was calculated between 350 and 550 ms after cue onset.

To assess cue-related brain activity, a repeated-measures ANOVA with the within-factors cue type (F-cue, R-cue), and valence (angry, neutral) was calculated for the above-specified components and time-windows, comparing across conditions average event-related activity within the frontal and parietal electrode groups specified above.

## Results

### Recognition rate

**FIGURE 2:**
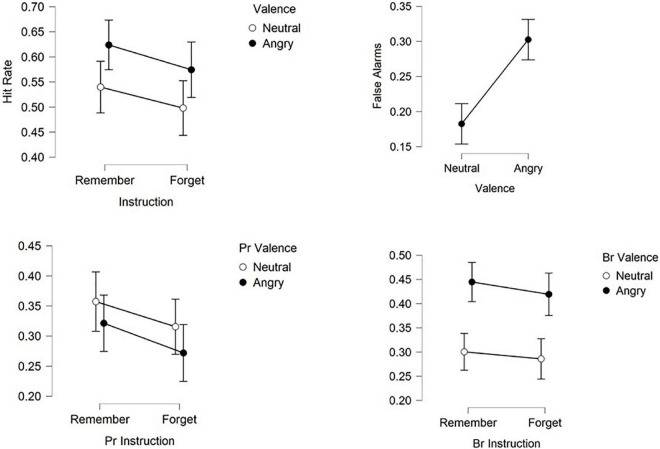
Hit rates, false alarms, recognition accuracy (Pr) and recognition bias (Br) for angry and neutral faces previously designated to be forgotten or to be remembered. Shown are means ± confidence intervals.

Mean recognition scores are displayed in Table 1 and plotted in Figure 2.

**TABLE 1:** Mean recognition performance for neutral and angry faces denoted as “to-be-forgotten” (F-items) or “to-be-remembered” (R-items).

	Neutral		Angry	
	F-items	R-items	F-items	R-items
**Hits**				
Rate	0.50 (0.04)	0.54 (0.04)	0.57 (0.03)	0.62 (0.03)
Reaction time	825 ms	827 ms	819 ms	840 ms
**Misses**				
Reaction time	870 ms	876 ms	871 ms	887 ms
**False alarms**				
Rate	0.18 (0.02)		0.30 (0.02)	
Reaction time	809 ms		826 ms	
**Correct rejections**				
Reaction time	853 ms		862 ms	

Statistical analysis on all previously presented items revealed that overall more items were subsequently recognized (hits = 0.56) than forgotten (misses = 0.44) [subsequent memory: *F*(1,24) = 5.73, *p* < 0.05, η_*p*_^2^ = 0.19, 1-β = 0.64]. Also, interactions between subsequent recognition and valence [*F*(1,24) = 5.523, *p* < 0.05, η_*p*_^2^ = 0.19, 1-β = 0.64] as well as between recognition and instruction [*F*(1,24) = 5.152, *p* < 0.05, η_*p*_^2^ = 0.18, 1-β = 0.41] were found. Based on these global effects a further ANOVA was calculated separately for recognized items (hits).

Analysis of hits revealed enhanced recognition of angry faces compared to neutral ones [valence: *F*(1,24) = 5.152, *p* < 0.05, η_*p*_^2^ = 0.18, 1-β = 0.41] and of R-items compared to F-items [instruction: *F*(1,24) = 5.523, *p* < 0.05, η_*p*_^2^ = 0.19, 1-β = 0.64]. Directed forgetting occurred for both angry and neutral expressions as reflected in the absence of an interaction between valence and instruction [*F*(1,24) = 0.037, *p* = 0.85, η_*p*_^2^ = 0.002, 1-β = 0.05].

Statistical analysis of responses concerning distractor items found more correct rejections than false alarms [*F*(1,24) = 154.912, *p* < 0.001, η_*p*_^2^ = 0.866, 1-β = 1] as well as an interaction between response and valence [*F*(1,24) = 36.883, *p* < 0.001, η_*p*_^2^ = 0.606, 1-β = 0.99]. A paired *t*-test on false alarms with the factor valence revealed elevated false alarms for faces with angry expressions compared to neutral expressions as reflected by a main effect of valence [*t*(24) = −6.073, *p* < 0.001, *d* = −1.25, 1-β = 0.99].

### Discrimination accuracy and bias

Simultaneously taking into account hits and false alarms and comparing the effects of instruction and facial expression on discrimination accuracy Pr and bias Br in recognition performance revealed an effect of instruction [*F*(1,24) = 5.152, *p* < 0.05, η_*p*_^2^ = 0.177, 1-β = 0.39] with better recognition for remember than forget items, but no effect of expression **[***F*(1,24) = 2.03, *p* > 0.1, η_*p*_^2^ = 0.08, 1-β = 0.11] on recognition accuracy. Instruction and expression did not interact **[***F*(1,24) = 0.037, *p* > 0.5, η_*p*_^2^ = 0.002, 1-β = 0.05].

Responses were found to be biased toward angry faces [*F*(1,24) = 19.488, *p* < 0.001, η_*p*_^2^ = 0.488, 1-β = 0.99] and bias was higher for remember than for forget faces **[***F*(1,24) = 4.696, *p* < 0.05, η_*p*_^2^ = 0.164, 1-β = 0.35] without interaction **[***F*(1,24) = 0.26, *p* > 0.5, η_*p*_^2^ = 0.01, 1-β = 0.05]. Figure 2 summarizes recognition performance.

### Reaction times

Statistical analysis of reaction times revealed a significant effect of recognition [*F*(1,24) = 45.84, *p* < 0.001; η_*p*_^2^ = 0.66, 1-β = 1] reflecting shorter reactions for recognized (hits: 828 ms) items compared to forgotten items (misses: 876 ms). An ANOVA with the factors response (correct rejections, false alarms) on the distractor data showed that correct rejections took longer than false alarms [*F*(1,24) = 9.29, *p* < 0.01; η_*p*_^2^ = 0.28; 1-β = 0.85]. Separate ANOVA on correct rejections and false alarms with the factor valence did not yield any significant results.

### EEG data

#### Face presentation

**FIGURE 3:**
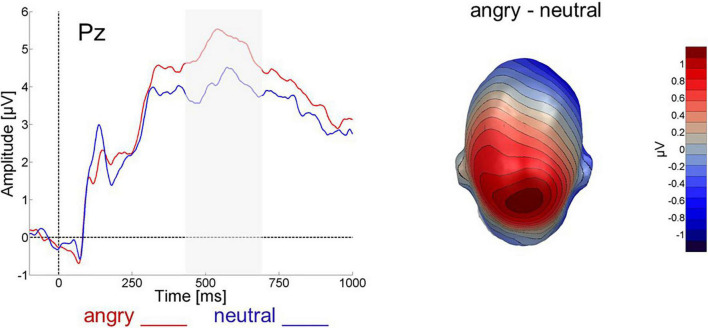
Face-evoked ERP for angry (red) and neutral (blue) faces at electrode Pz and difference topography for angry minus neutral faces across all 64 electrodes between 450 and 700 ms after face onset.

An ANOVA with the factor valence (angry, neutral) revealed that over the parietal electrode group angry expressions (mean = 0.229, SD = 0.512) elicited more positive-going activity than neutral faces (mean = 0.053, SD = 0.399) between 450 and 700 ms after picture onset [*F*(1,24) = 6.436, *p* < 0.05, η_*p*_^2^ = 0.211, 1-β = 0.51], see Figure 3].

#### Cue presentation

##### Frontal P2

**FIGURE 4:**
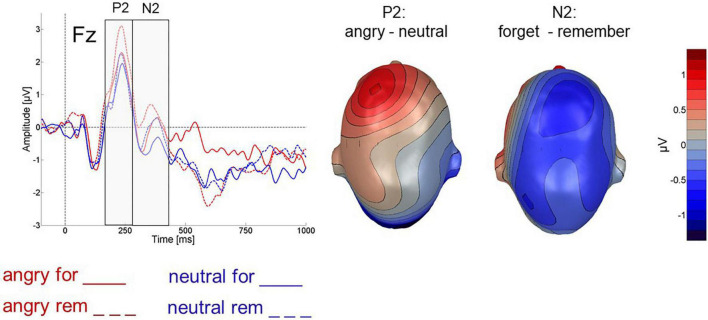
**Left:** Cue-evoked ERP for cues following angry (red) and neutral (blue) faces at electrode Fz. **Right:** Difference topography for angry minus neutral faces across all 64 electrodes for the P2 and N2 time-windows highlighted on the left.

Figure 4 illustrates frontal P2 and N2 ERP effects. Mean ERP amplitudes for the analyzed electrode groups are detailed in Table 2. In the frontal P2 component, cues following angry faces elicited a larger P2 than cues following neutral faces [valence: *F*(1,24) = 6.751; *p* < 0.05, η_*p*_^2^ = 0.22, 1-β = 0.56]. However, P2 amplitude did not vary with the type of cue [instruction: *F*(1,24) = 1.745; *p* > 0.1, η_*p*_^2^ = 0.068, 1-β = 0.1] and the two factors did not interact [valence X instruction: *F*(1,24) = 1.503; *p* > 0.1, η_*p*_^2^ = 0.059, 1-β = 0.08].

**TABLE 2:** Means and standard deviations for P2 and N2 ERPs elicited by cues following neutral (ntr) or angry faces.

		Ntr F	Ntr R	Angry F	Angry R		Ntr F	Ntr R	Angry F	Angry R
	P2					N2				
Mean		1.386	1.366	1.603	2.024		−0.120	0.198	0.195	0.563
SD		1.541	1.926	1.623	2.068		1.598	1.397	1.383	1.825
Min		1.117	−1.105	−2.276	−1.416		−2.430	−2.575	−2.794	−2.115
Max		3.722	5.565	5.269	5.455		5.872	2.711	2.928	5.363

F denotes forget cues, R denotes remember cues. Means indicate average activity across a group of electrodes and all participants.

##### Frontal N2

In the frontal N2 (280 – 400 ms after cue onset), F-cues elicited a more pronounced negativity than R-cues which was reflected in a main effect of instruction [*F*(1,24) = 4.960; *p* = 0.036, η_*p*_^2^ = 0.171, 1-β = 0.37, see Figure 4]. In tendency, cues following neutral faces elicited more negative-going ERP than cues following angry faces (valence: [*F*(1,24) = 3.759; *p* < 0.1, η_*p*_^2^ = 0.135, 1-β = 0.25]. The effects of instruction and face valence did not interact [instruction x valence: *F*(1,24) = 0.018; *p* > 0.5, η_*p*_^2^ = 0.007, 1-β = 0.05].

##### Late parietal positivity

**FIGURE 5:**
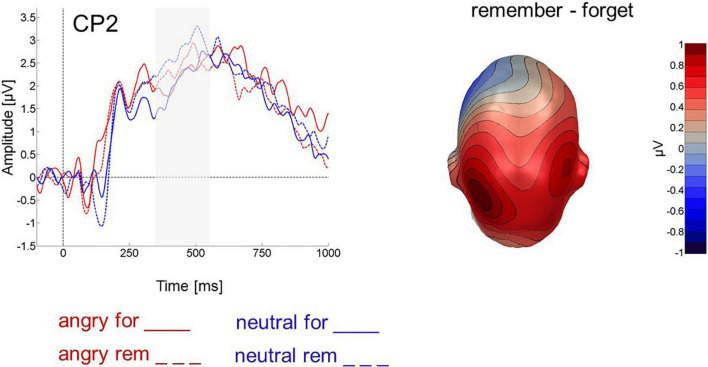
Cue-evoked ERP for cues following angry (red) and neutral (blue) faces at centro-parietal electrode CP2. Forget cues are solid lines, remember cues are dashed. Right panel shows difference topography for remember minus forget cues across all 64 electrodes between 350 and 550 ms after cue onset.

An ANOVA with the factors instruction (F-cue, R-cue) and valence (angry, neutral) assessed parietal activity between 350 and 550 ms after cue onset and revealed that R-cues elicited more positivity [instruction: *F*(1,24) = 9.590, *p* < 0.01, η_*p*_^2^ = 0.128, 1-β = 0.233, Figure 5] and so did cues following neutral faces compared to those following angry faces [valence: *F*(1,24) = 6.079, *p* < 0.05, η_*p*_^2^ = 0.046, 1-β = 0.07]. The interaction was not significant [instruction X valence: *F*(1,24) = 0.152, *p* > 0.5, η_*p*_^2^ = 0.002, 1-β = 0.05]. Table 3 details mean late cue-evoked ERP amplitudes for the analyzed electrode groups.

**TABLE 3:** Means and standard deviations for LPP (late parietal positivity) and LFP (late frontal positivity) ERPs elicited by cues following neutral (ntr) or angry faces.

		Ntr F	Ntr R	Angry F	Angry R		Ntr F	Ntr R	Angry F	Angry R
	LPP					LFP				
Mean		−0.032	0.235	−0.143	0.063		−0.264	−0.267	−0.084	−0.508
SD		0.657	0.597	0.708	0.595		0.489	0.665	0.579	0.565
Min		−1.563	−1.057	−3.009	−1.380		−1.341	−1.740	−1.393	−1.839
Max		1.274	1.407	0.578	1.349		0.783	1.932	1.481	0.628

F denotes forget cues, R denotes remember cues. Means indicate average activity across a group of electrodes and all participants.

##### Late frontal positivity

**FIGURE 6:**
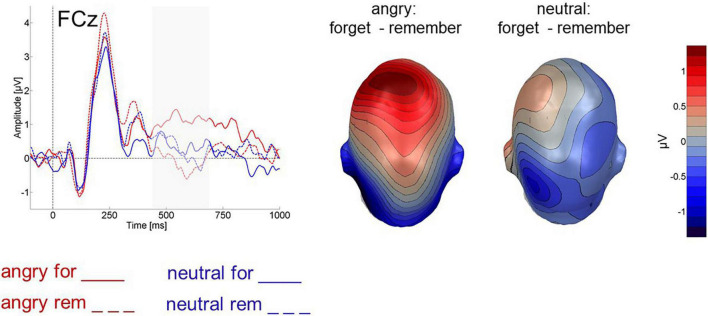
Cue-evoked ERP for cues following angry (red) and neutral (blue) faces at fronto-central electrode FCz. Forget cues are solid lines, remember cues are dashed. Right panel shows difference topographies for forget minus remember cues, separately for cues following angry and neutral faces, across all 64 electrodes between 450 and 700 ms after cue onset.

An ANOVA with the factors instruction (F-cue, R-cue) and valence (angry, neutral) assessed frontal activity between 450 and 700 ms after cue onset. F-cue related activity was more positive than R-cue related activity [instruction: *F*(1,24) = 5.539, *p* < 0.05, η_*p*_^2^ = 0.188, 1-β = 0.44, Figure 6]. There was no difference in amplitudes between cues following angry and neutral faces *per se* [valence: *F*(1,24) = 0.211, *p* > 0.5, Figure 6], but an interaction between valence and instruction [*F*(1,24) = 6.437, *p* < 0.05, η_*p*_^2^ = 0.211, 1-β = 0.53, Figure 6] revealed that this enhanced positive-going activity during the F-Cue was in particular present after angry faces. Specifically, only for angry faces was the ERP elicited by the F-Cue more positive than the ERP elicited by the R-Cue (*t*(24) = 3.449, *p* < 0.01, Bonferroni-Holm corrected). None of the other pairwise comparisons approached significance (*p* > 0.1, Bonferroni-Holm corrected).

##### Relationship between behavior and ERP data

As in previous reports (Hauswald et al., 2011; Schindler and Kissler, 2018; Alfonso and Menor, 2021), we correlated the magnitude of the directed forgetting effect (remember minus forget) with the respective ERP amplitudes during face and cue processing, but failed to find a clearly significant effect. There was, however, a close-to-significant correlation between (*r* = 0.39, *p* = 0.054) between the directed forgetting effect for angry faces (hits remember – hits forget) and F-cue evoked potentially relevant late frontal ERP positivity following angry faces. No other correlation approached significance.

## Discussion

This study investigated behavioral and electrophysiological mechanisms of item-method DF for angry and neutral faces. Behaviorally, both hit and false alarm rates were higher for angry than for neutral faces, in line with the notion that “better” recognition of emotional items can be contributed to by both recollective processes (Johansson et al., 2004) and response biases in favor of emotional items (Windmann and Kutas, 2001; Dougal and Rotello, 2007). Here, the effect was primarily due to a response bias toward angry faces. Crucially, a DF effect was found for both facial expressions and it was present for both recognition accuracy and response bias, resulting in reduced recognition accuracy and response bias for to-be-forgotten faces. As hypothesized, during stimulus presentation angry faces elicited larger parietal LPPs than neutral faces. When the cue was presented, F-cues elicited larger N2 than R-cues. Conversely, R-cues induced larger parietal LPPs than forget cues. Both these findings were in line with our theoretical expectations. Notably, forget cues following angry faces elicited a late frontal positivity that was absent for forget cues following neutral faces. This finding was partly in line with our hypotheses as we had expected a late frontal positivity to be elicited by F-Cues, but had no expectations regarding its modulation by the expression of the previous face.

We also observed several other effects of facial expression on ERPs for which we had no firm *a priori* expectation: P2 was larger following angry than neutral faces whereas cues following neutral faces elicited larger LPPs than cues following angry faces, regardless of implied instruction.

These results replicate several findings from the literature and add new evidence. They demonstrate that, within one study, different neural mechanisms can give rise to the item-method DF effect. Behaviorally, data are in line with the findings by Quinlan and Taylor (2014) who found similar magnitude item-method DF for angry and neutral faces. Tay and Yang (2017), by contrast, reported angry faces to be more resistant to directed forgetting than happy or neutral ones. Given that in Tay and Yang’s study emotional expressions were presented in the learning phase and recognition was tested on the same faces with a neutral expression, whereas Quinlan and Taylor (2014) and the present study tested recognition of identical stimuli, specific experimental parameters and in particular task difficulty may influence whether item-method DF is found for angry faces.

Regarding ERPs, confirming previous results, angry faces induced larger parietal LPPs upon presentation than did neutral faces (Schupp et al., 2004; Recio et al., 2011). Also in line with previous results, the remember cue induced a larger parietal LPP than the forget cue. This is in accord with the selective rehearsal account of directed forgetting and replicates similar previous findings for pictures (Hauswald et al., 2011; Schindler and Kissler, 2018) and words (Brandt et al., 2013; Alfonso and Menor, 2021), extending them to faces. This effect was independent of the expression of the preceding face. In the studies by Brandt et al. (2013) as well as in Alfonso and Menor’s (2021), by contrast, R-cue related LPP amplitude was higher following emotional than neutral words. As a tentative explanation and as suggested by Brandt et al. (2013), visually presented word stimuli may be easier to selectively rehearse than pictorial stimuli, because they have access to both phonological and visual working memory (Baddeley, 2003) and can be rehearsed *via* inner speech. Pictorial stimuli, including faces, may decay faster, potentially leaving less time for differential rehearsal of emotional versus neutral stimuli. Faces are structurally very similar and generally more difficult to remember than words or more complex scenes which is also reflected in the presently lower recognition performance than in previous DF studies using words (Bailey and Chapman, 2012; Brandt et al., 2013; Gallant and Dyson, 2016; Alfonso and Menor, 2021) or pictures (Hauswald et al., 2011; Yang et al., 2012; Schindler and Kissler, 2018). Still, the present behavioral data are quite similar to Quinlan and Taylor’s (2014) or Tay and Yang’s (2017). Surprisingly, we also found cue-related LPPs to be more positive-going following neutral than angry faces, regardless of the presented cue. If this is not an accidental finding, it may reflect that neutral faces are even harder to keep in memory than angry ones and therefore require the allocation of more resources throughout.

We also analyzed several cue-related frontal ERP components thought to indicate attention allocation and inhibitory processing. The frontal P2 that other studies reported to index attention deployment toward the R-cue (Gao et al., 2016; Schindler and Kissler, 2018; Alfonso and Menor, 2021) was larger following angry than neutral faces. Cue-type did not significantly affect its magnitude. Given that previous studies suggested the P2 to reflect relevance for working memory storage (Getzmann et al., 2018), the natural relevance of emotional expressions may at least in some cases override the task-induced relevance represented by the R-cue.

By contrast, the frontal N2 was sensitive to the meaning of the cue as reflected in more negative-going N2 waves for F-cue than R-cues. This stands in agreement with other research that found larger N2 for F- than R-cues in item-method DF (Gao et al., 2016; Schindler and Kissler, 2018). Considering this component as a neural index of inhibitory processing across various cognitive domains, our data support the view that inhibitory processes are triggered in the item-method DF paradigm. In tendency, the frontal N2 was larger following neutral than angry faces which would contrast with the findings by Yang et al. (2012) who reported a larger N2 elicited by F-cues following negative pictures, but since this effect was not clearly significant, we refrain from further interpretation. Likewise, Patrick et al. (2015) found no difference between N2 amplitude for F-cues following negative or neutral words. At any rate, the finding of F-cues eliciting larger N2 than the R-cue in the item-method DF paradigm seems relatively robust (see also Gao et al., 2016). However, the data beg questions regarding the functional relevance of the N2 in item-method DF: Does it reflect a system alert to the need to inhibit a subsequent processing stage or does it already represent the inhibition itself? Evidence from the number-letter task suggests that the N2 reflects attentional inhibition of working memory access for irrelevant items in order to shield the processing of relevant stimuli (Getzmann et al., 2018). Thus, the N2 could already reflect inhibition of to-be-forgotten items, regardless of their affective significance. On the other hand, using a “count/no count task,” Zhang et al. (2021) recently failed to observed N2 modulation by inhibition requirements in the “no count” condition. Instead, they observed a frontal positivity (termed frontal P3) increase in the “no count” compared to the count task which was interpreted as reflecting conflict resolution.

Similarly, we also found a frontal positivity in response to forget cues. However, it occurred only following angry faces and was absent for forget cues following neutral items. In item-method DF, relatively late frontal positivities elicited by forget cues have been reported previously (Paz-Caballero et al., 2004; Hauswald et al., 2011; Brandt et al., 2013; Gallant and Dyson, 2016; Alfonso and Menor, 2021), although typically not differing between cues following negative and neutral items (e.g., Hauswald et al., 2011; Brandt et al., 2013; Gallant and Dyson, 2016). To our knowledge only Alfonso and Menor (2021) report a marginal emotion effect on the frontal positivity, with a somewhat larger frontal positivity following neutral rather than negative words, which is descriptively also present in Hauswald et al. (2011) but opposite of the present pattern. Such frontal positivities have been linked to inhibitory processes involved in item-method directed forgetting. Their magnitude had been shown to correlate positively with the directed forgetting effect for pictures (Hauswald et al., 2011), indicating more forgetting with higher frontal positive ERPs. By contrast, recently the frontal positivity has also been found to correlate with higher hit rate for forget items (Schindler and Kissler, 2018). Neither Gallant and Dyson (2016) nor Alfonso and Menor (2021) report further correlative evidence that might help elucidate the function of this positivity. Here, we found a trend-level correlation between the directed forgetting effect for angry faces (hit rate remember angry minus hit rate forget angry) and the frontal positive amplitude which might agree with an inhibitory account. On the other hand, the relationship was not clearly significant and nor was any other meaningful correlation. Interestingly, recent evidence from a working memory Stroop paradigm (Wang et al., 2021) shows a functional dissociation between N2 and P3. The authors interpreted the frontal N2 in terms of conflict monitoring and the frontal P3 in terms of conflict resolution. If so, neutral and angry faces would invoke conflict monitoring to a similar extent, but more conflict resolution would be induced by angry faces cued to be forgotten.

Several previous studies have taken advantage of subsequent memory analysis and old-new effects, *post-hoc* dividing the items into incidentally (R-items) versus intentionally (F-items) subsequently forgotten versus analogously classified remembered items to assess the mnemonic consequences of the remember versus the forget instructions (e.g., Ullsperger et al., 2000; Nowicka et al., 2009) or to assess the functional significance of ERPs during the encoding phase (Gao et al., 2019). Indeed, such an approach could turn out to be very helpful in clarifying the role of the observed effects and we had aimed for it in the present study. However, because we had only 30 trials per stimulus category and a hit rate between 50 and 60%, we would have had fewer than 15 useable trials in each cell for the subsequent memory analysis, preventing us from presenting such an analysis. In general, those analyses are complicated by the need to have a relatively even distribution of subsequently remembered and forgotten trials (which can be achieved with faces, because face recognition memory is poorer than the one for scenes or words) and still a reasonable number of trials when further looking at emotion modulations (which was our major obstacle). Future studies should aim for such analyses. Since the effects of item-method DF are generally thought to be generated predominantly in the encoding phase, we still believe that the presented ERP effects already capture an important part of the underlying mechanisms.

Although we obtained clearly significant DF effects on the behavioral level, at 4–5% reduction in recognition, they were smaller than typically obtained with pictures (>10%). Such smaller effects with less variance leave less room for potentially interesting correlations. Nevertheless, the data pattern indicates that the same behavioral DF effect might be achieved *via* different neural mechanisms, depending on the emotional content of the preceding stimulus. Although F-cues in general were found to elicit a N2, DF of neutral faces further recruited processes that seem consistent with selective rehearsal of remember items. By contrast, item-method DF of angry faces involved a larger LPP during pre-Cue stimulus presentation as well as an additional late frontal positivity. The frontal positivity might be generated in response to stronger interference caused by the more pronounced incidental processing of preceding angry faces.

While our results suggest at least two ways to generate DF, one by selective rehearsal and another one by potentially inhibitory processes, they beg the question as to why previous studies did not observe such a content-dependence. To allow for a meaningful ERP analysis, a rather high number of faces was used in this study. Therefore, structural similarity of the many faces used in the present study may not have resulted in much automatic encoding particularly of the neutral faces, such that they did not require much additional interference resolution. By contrast, the angry faces were immediately processed more deeply, as evidenced by their larger LPP. Indeed, without wanting to overstretch the implications of this observation, there was a positive correlation between the LPP elicited by the angry faces and the late frontal positivity evoked by the subsequent F-cue (*r* = 0.46, *p* < 0.05) which was not present for neutral faces (*r* = 0.17, *p* = 0.4) or for the N2 component (angry: *r* = 0.1, *p* = 0.6; neutral: *r* = 0.002, *p* = 0.95). This pattern may underscore a relationship between initial item processing and subsequent F-cue evoked frontal positivity. If so, experimental manipulation of encoding difficulty, perhaps realized *via* manipulating item number, distinctiveness and presentation time should likewise affect the presence and magnitude of the frontal positivity in a similar way as emotional content. Accordingly, Nowicka et al. (2011), in an fMRI study that used very brief picture presentation, found directed forgetting for both emotional and neutral items. However, forgetting of emotional pictures elicited frontal activation that was absent for neutral pictures. Hauswald et al. (2011), by contrast, presented pictures for 2s and found a frontal positivity for both neutral and negative pictures, although it was descriptively larger for neutral pictures. The absence of directed forgetting for negative pictures in Hauswald et al.’s study was thought to be due to a combination of their enhanced pre-cue stimulus processing and insufficient inhibitory processing. Here, we found similar magnitude DF for neutral and angry faces along with a larger frontal positivity elicited by forget cues following angry but not neutral faces. Although we cannot yet be sure about the functional significance of each component, we demonstrate that in item-method DF different neural processes can sub-serve the same behavioral outcome: Overall, the present study reveals behavioral directed forgetting for both angry and neutral faces, but demonstrated different neural mechanisms contributing to this effect: One, predominantly recruited for neutral faces, that is most consistent with conflict monitoring followed by selective rehearsal and a second one that reveals an additional process mediated by the frontal brain which may reflect conflict resolution instead of selective rehearsal.

### Limitations

Although the present study replicates some established findings regarding the electrophysiology of item-method DF and adds new evidence regarding mechanisms of directed forgetting of angry versus neutral faces, several limitations need to be kept in mind: Firstly, a larger and gender balanced sample would have been desirable. At 25 participants, the present sample lies well within what has been typical for the field. For instance, Hauswald et al. (2011) had 19, Yang et al. (2012) and Brandt et al. (2013) had 17 participants, Patrick et al. (2015) had 20 in the healthy control group, Gao et al. (2016) had 23, Ye et al. (2019) had 24. Larger studies include Bailey and Chapman (2012), Gallant and Yang (2014), and Alfonso and Menor (2021), who had 32, 36, and 33 participants, respectively. Whereas the present as well as previous samples are clearly big enough to show standard main effects of directed forgetting, some of the neurophysiological effects might have benefited from more experimental power. Moreover, a gender-balanced sample would have been desirable. The present participants were recruited as a convenience sample among undergraduate psychology students which tend to be predominantly female. However, we recognize that regarding emotion modulation of the present effects, gender differences may exist, although they are likely to be small (for review see e.g., Thompson and Voyer, 2014). Still, for list-method DF of spoken words, Yang et al. (2013) showed reduced effects in females and for lists consisting of words spoken by females. Thus, future studies should examine whether similar effects exist in item-method DF. Finally, given that across studies some basic effects of item-method DF and the electrophysiological mechanisms involved are quite robust whereas others seem more variable, more systematic assessment of effects on ERP components in item-method DF of individual experimental parameters such as emotional arousal, stimulus and cue presentation times or post-cue rehearsal time seems warranted (see also Titz and Verhaeghen, 2010 for a meta-analysis of various factors impacting on behavioral DF effects).

## Conclusion

In conclusion, this study demonstrates on the behavioral level equivalent item-method directed forgetting for angry and neutral faces, as well as an LPP elicited by the R-cue likely reflecting selective rehearsal and an N2 potential induced by the F-cue which seems to signal conflict and inhibition of further processing. These effects do not differ depending on the expression of the presented faces. However, differing mechanisms are reflected in a number of positive ERPs: Angry faces elicit larger LPP during face presentation. During cue presentation, the P2 following angry faces is bigger than the one following neutral faces and selectively for F-cues following angry faces a larger late frontal positivity potentially reflecting conflict resolution is observed. Thus, this study demonstrates how different neural mechanisms can sub-serve the same behavioral outcome.

## Data availability statement

The raw data supporting the conclusions of this article will be made available by the authors, without undue reservation.

## Ethics statement

The studies involving human participants were reviewed and approved by the University of Konstanz Ethics Committee. The patients/participants provided their written informed consent to participate in this study.

## Author contributions

JK designed the experiment. AH ran the experiment. AH and JK analyzed the data and wrote the manuscript. Both authors contributed to the article and approved the submitted version.
